# Supplementary figures and images for: Integrated annotation prioritizes metabolites with bioactivity in inflammatory bowel disease
Source: Mol Syst Biol. 2024 Mar 11;20(4):338–61. doi: 10.1038/s44320-024-00027-8 (PMC10987656; doi:10.1038/s44320-024-00027-8)

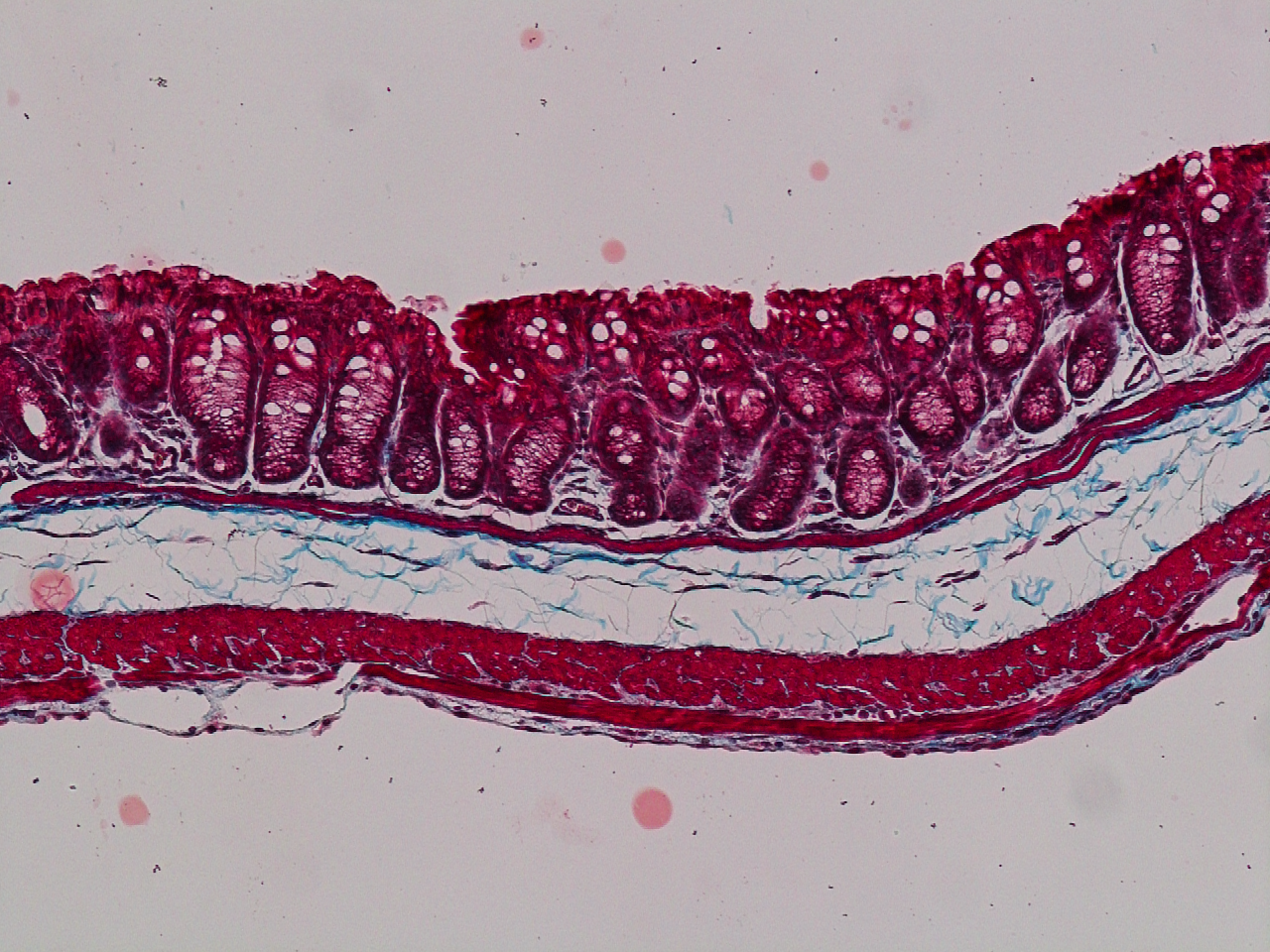

Supplement: Supplementary file 11 — Source Data Fig. 5 [file 44320_2024_27_MOESM11_ESM.zip › Source_data/Fig5F_Fig5H_Histology/Fig5H_NR_original.tif]

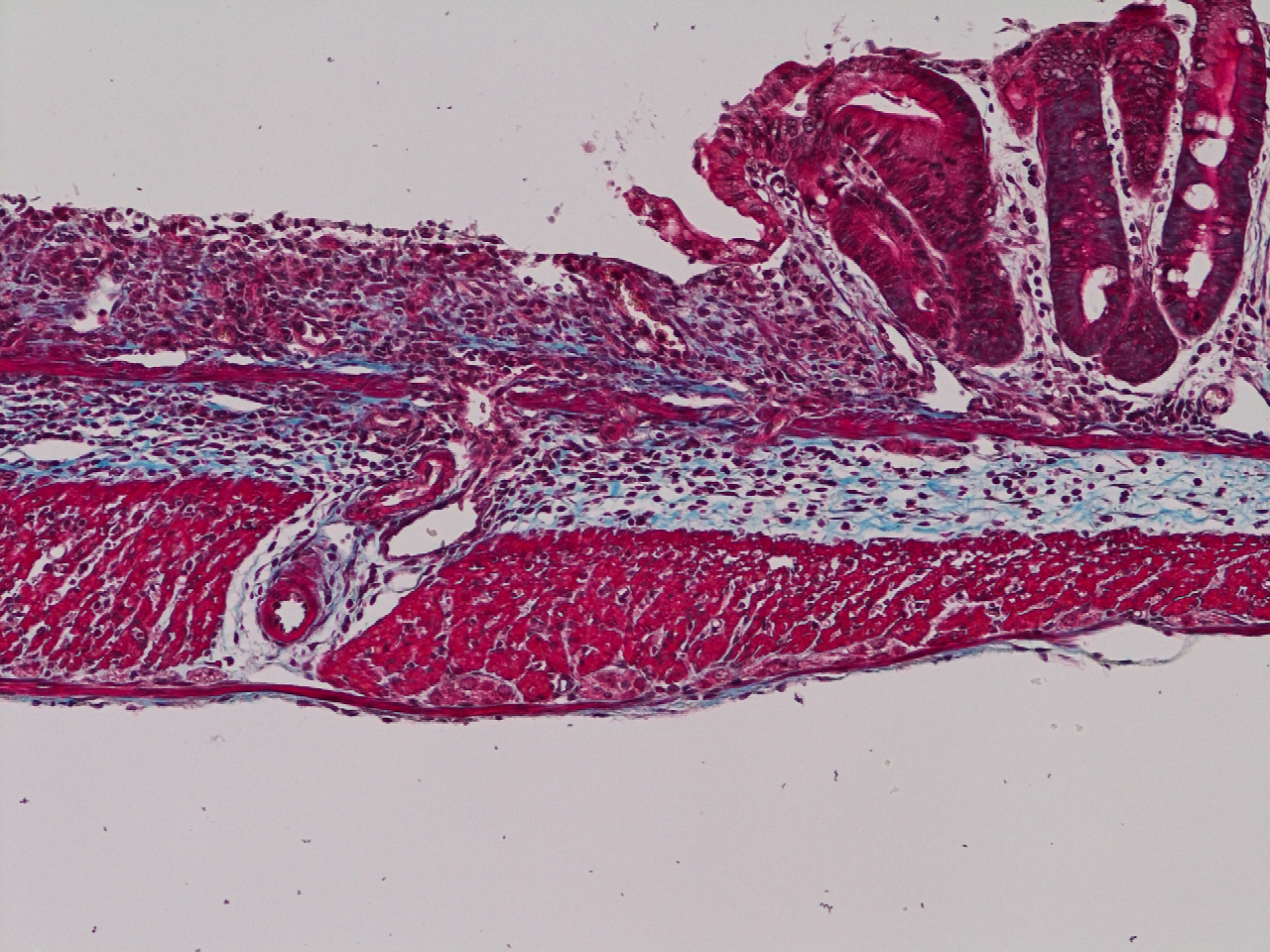

Supplement: Supplementary file 11 — Source Data Fig. 5 [file 44320_2024_27_MOESM11_ESM.zip › Source_data/Fig5F_Fig5H_Histology/Fig5H_PBS_original.tif]

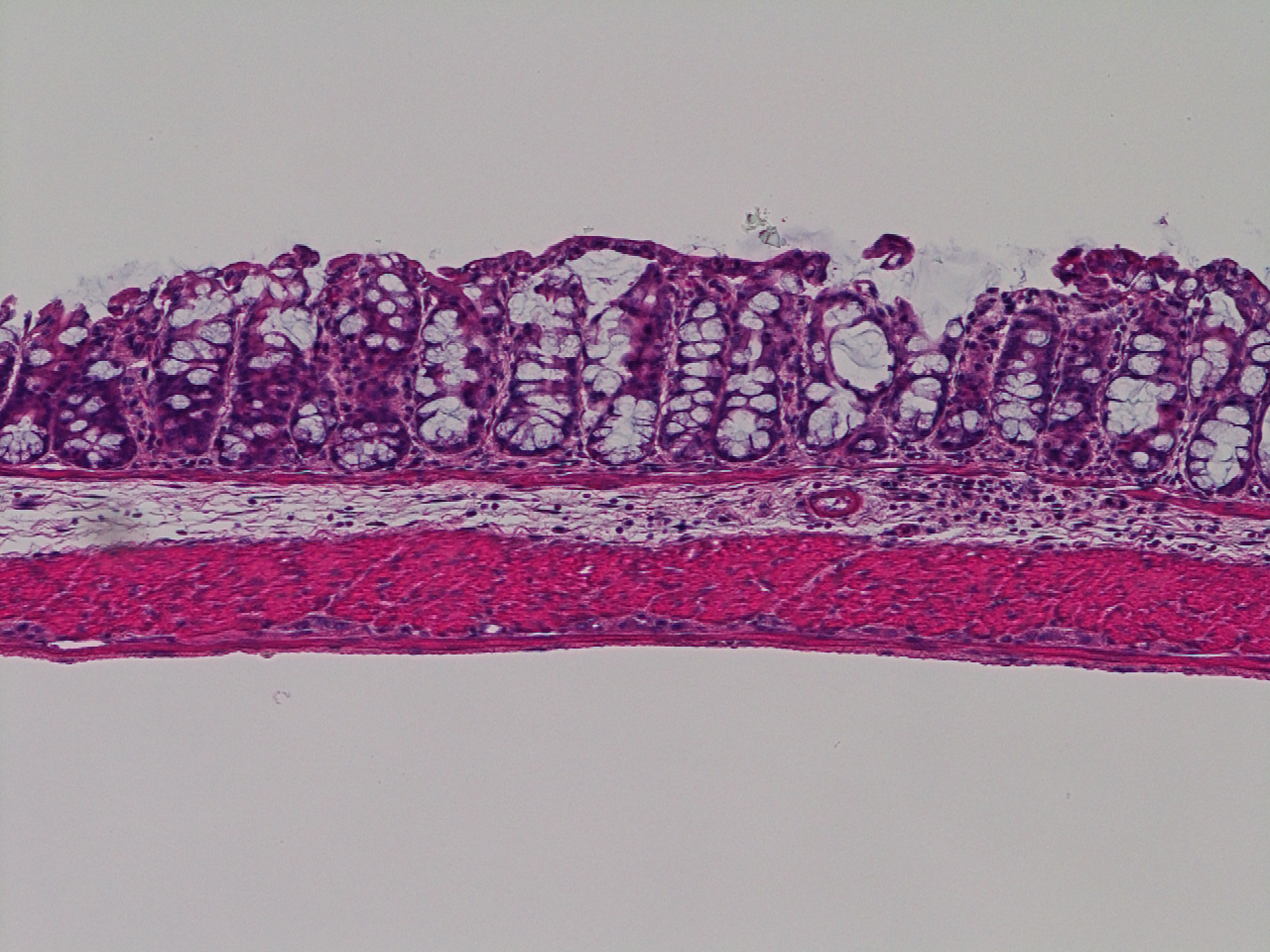

Supplement: Supplementary file 11 — Source Data Fig. 5 [file 44320_2024_27_MOESM11_ESM.zip › Source_data/Fig5F_Fig5H_Histology/Fig_5F_NR_original.tif]

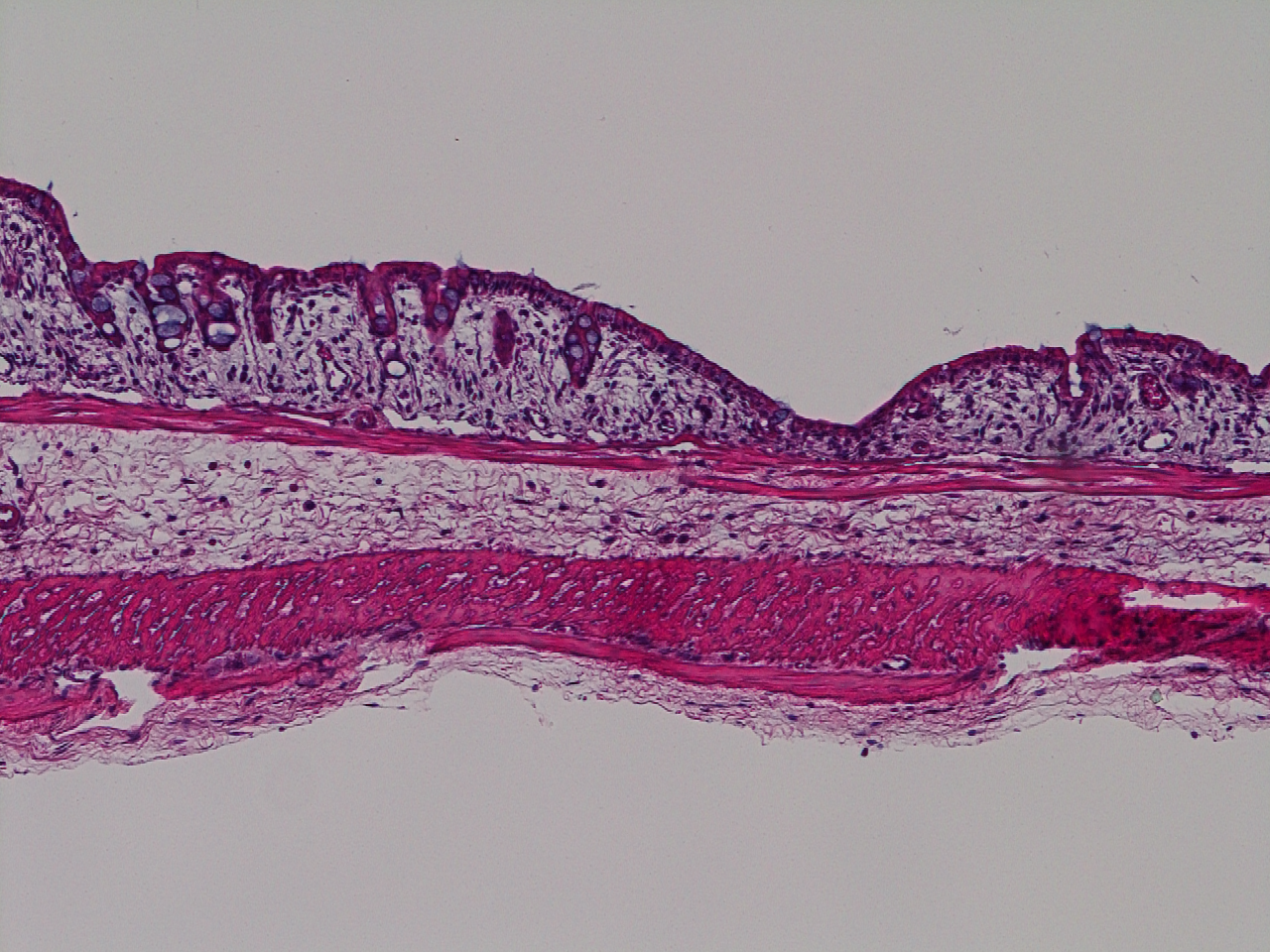

Supplement: Supplementary file 11 — Source Data Fig. 5 [file 44320_2024_27_MOESM11_ESM.zip › Source_data/Fig5F_Fig5H_Histology/Fig_5F_PBS_original.tif]
