# Supplementary material for: Integrated annotation prioritizes metabolites with bioactivity in inflammatory bowel disease
Source: Mol Syst Biol. 2024 Mar 11;20(4):338–61. doi: 10.1038/s44320-024-00027-8 (PMC10987656; doi:10.1038/s44320-024-00027-8)
Supplement: Supplementary file 13 — Expanded View Figures [file 44320_2024_27_MOESM13_ESM.pdf]

Expanded View Figures

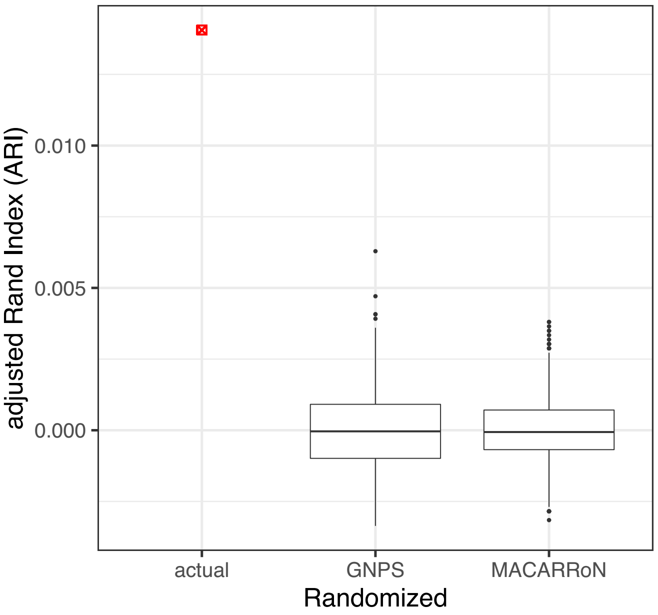

Figure EV1. Adjusted Rand Index (ARI) values for the overlap between actual and randomized MACARRoN modules and GNPS clusters (molecular networks).

Overlap between GNPS cluster and MACARRoN module assignments of 1311 features in the sputum metabolomes was determined using ARI. GNPS cluster and MACARRoN module assignments were then shuffled and overlap with actual MACARRoN modules and GNPS clusters respectively was calculated. This was performed 1000 times each for both GNPS and MACARRoN. The ARI obtained by comparing actual assignments was significantly higher than ARIs where one assignment was randomized. Bounds of boxplots show 1st quartile and 3rd quartile and the line inside the box is the median.

 © The Author(s)

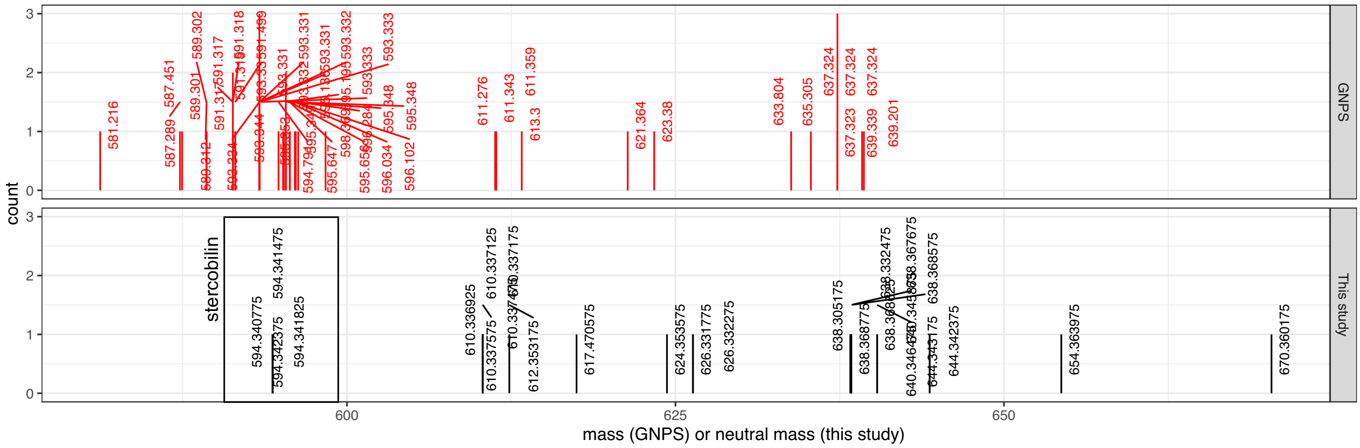

Figure EV2. Masses of highly prioritized features in modules 10 and 85, and compounds in stercobilin molecular networks across 11 MassIVE datasets.

(bottom) Shown are the highly prioritized features in modules 10 and 85 with $|\Delta m/z|$ <100 w.r.t stercobilin. (top) Compounds that were directly connected to stercobilin or urobilin in molecular networks within the aforementioned mass-difference shown. The tight cluster of masses represents masses associated with stercobilin in these molecular networks. Count represents the number of times a particular mass was observed.

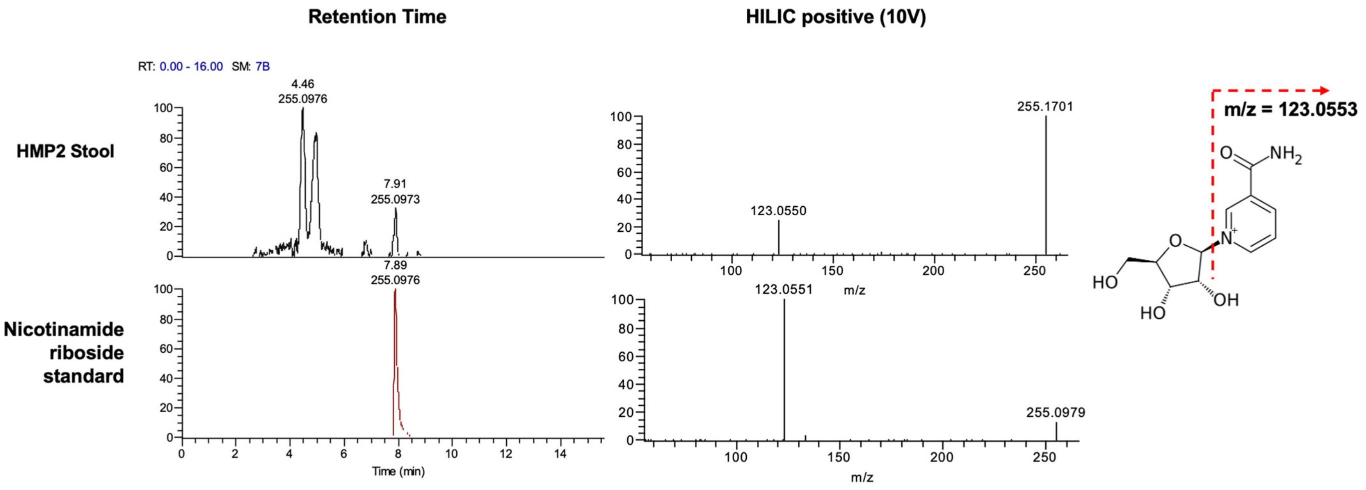

Figure EV3. Identification of feature representing nicotinamide riboside in the HMP2.

Retention time and fragmentation pattern on HILIC-positive are shown for HMP2 feature and nicotinamide riboside standard.

 © The Author(s)
